# Comparative study in infertile couples with and without *Chlamydia trachomatis* genital infection

**DOI:** 10.1186/s12978-016-0271-4

**Published:** 2017-01-13

**Authors:** Yuanchang Zhu, Biao Yin, Tonghua Wu, Lijun Ye, Chunmei Chen, Yong Zeng, Yaou Zhang

**Affiliations:** 1 School of Life Sciences, Tsinghua University, Beijing, 100000 China; 2 Key Lab in Healthy Science and Technology, Division of Life Science, Graduate School at Shenzhen, Tsinghua University, Shenzhen, 518000 China; 3 Central South University, the State Key Laboratory of Medical Genetics of China, Changsha, 410000 China; 4 Shenzhen Key Laboratory for Reproductive Immunology of Preimplantation, Shenzhen Zhongshan Institute for Reproduction and Genetics, Fertility Center, Shenzhen Zhongshan Urology Hospital, Shenzhen, 518000 China

**Keywords:** *Chlamydia trachomatis*, Subfertile, Epidemiology, Assisted reproduction technique, Clinical outcome

## Abstract

**Background:**

*Chlamydia*, caused by the bacterium *Chlamydia trachomatis(C. trachomatis)*, is the most common sexually transmitted disease. The incidence is not clear due to the asymptomatic nature of early stage of infections. The incidence of *Chlamydia* has not been fully investigated in the Chinese Han population. Since chronic infection with can *C. trachomatis* can lead to infertility in males and females, it is important to determine the impact of infection on clinical outcomes. The aim of this study is to explore the epidemiology of *C. trachomatis* in subfertile couples and to determine whether infections will adversely affect clinical outcomes after assisted reproduction technique (ART) treatment.

**Methods:**

Subfertile patients (*n* = 30760) were screened in the research for *C. trachomatis* in our center from January 2010 to December 2014. *C. trachomatis*-specific DNA was detected by Taq-man PCR from semen or swabs from the urethral, endocervix or vaginal. The control group consisted of 1140 subfertile patients without *C. trachomatis* infection. The prevalence and characteristics of *C. trachomatis* were identified for subfertile couples and clinical outcomes were collected and analyzed. A retrospective study was performed.

**Results:**

Nine hundred and seventy patients were diagnosed with *C. trachomatis* infection, and the overall prevalence was 3.15% in the most recent five years, with a yearly increasing. The incidence was a higher in the second half of the year (3.40%) compared to the first half (2.69%). The age group with the highest-risk of infection with *C. trachomatis* was between 26 to 35 years old, and in about one third of the couples, both partners were infected. The basic parameters and clinical outcomes were not statistically significant between different the groups (*P* > 0.05), even though some minor data were different (*P* < 0.05).

**Conclusions:**

*C. trachomatis* is a common infection in subfertile people and it is essential to test for this organism in ART couples’ screening. This study identified no adverse on clinical outcomes after successful treatment of *C. trachomatis* infection, regardless of gender, age and number of *C. trachomatis* copies.

## Plain English summary

One of the most common bacterial sexually transmitted disease is *Chlamydia*, *caused by Chlamydia trachomatis* (*C. trachomatis)*, the exact incidence of *Chlamydia* is unknown because the infection is often asymptomatic in the early stage. The incidence of *Chlamydia* it has not been fully investigated in the Chinese Han population. Since chronic infection with *C. trachomatis* can induce infertility in males and females. The aim of the study is to explore the epidemiology of *C. trachomatis* in subfertile couples and to determine adverse effects on clinical outcomes after assisted reproduction technique (ART) treatments.

A total of 30760 subfertile patients were tested for *C. trachomatis* in our center from January 2010 to December 2014. *C. trachomatis*-specific DNA was detected by Taq-man PCR. The prevalence and characteristics of *C. trachomatis* were identified for subfertile couples and clinical outcomes were collected and analyzed. A retrospective study was performed.

The overall prevalence of *C. trachomatis* was 3.15% in a recent five year periods, with a yearly increase. The incidence was slightly higher in the second half of the year (3.40%) compared to the first half of the year (2.69%). The age range with the highest-risk infection of of *C. trachomatis* was between 26 and 35 years, and in about one third of the couples, both partners had the infection. The main parameters and clinical outcomes were not statistically significant between the groups (*P* > 0.05), even though some minor data were different (*P* < 0.05).

The results of this ananlysis confirm that of *C. trachomatis* is common infection in subfertile people and it is essential to test for the presence of this microbe as part of ART couples’s screening. This study found no adverse effects on clinical outcomes after the infection was cured.

## Background

*Chlamydia trachomatis (C. trachomatis)*, an intracellular bacterium, is the cause of the most frequently sexually transmitted disease (STD), resulting in about 100 million new cases annually worldwide [1]. Previous studies have reported that the prevalence of *C. trachomatis* ranged from 1.7% to 17% among European women [2] and from 1%-40% among asymptomatic men with a longstanding history of couple infertility [3], depending on the method of diagnosis, social background, area of residence, occupation, gender, and among others. Approximately 50% males and 80% females with *Chlamydia* show no symptoms in the early stage of infection, consequently, many of these individuals remain undiagnosed and are often poorly or inadequately treated [4]. Therefore, the true prevalence of this infection is not clear, and may remain unknown for several years or even longer. Standard microbiologic tests were commonly used to diagnosis *C. trachomatis* infections in the past, but studies have proved that this method may fail to reveal the pathogen, owing to high-proportion false negative results [5]. Thus, the DNA amplification method is increasingly universal. This method has a high sensitivity (99%) and specificity (100%), and has been accepted as the gold standard for the identification of *C. trachomatis* infections. [6, 7].

Since *C. trachomatis* is often asymptomatic and treatment is often delayed, the infection is often transmitted to sexual partner(s) [8]. It can cause fallopian tube salpingitis, cervicitis, and pelvic inflammatory disease (PID), which in turn can cause infertility and ectopic pregnancy in women with chronic infections [9].

Research has shown that microbiologic tests done on semen revealed a high proportion (43%) of *C. trachomatis* -specific DNA in semen from men with azoospermia [10], suggesting that hronic progression of *C. trachomatis* in the male genital tract may lead to urethritis, epididymitis, and even obstructive azoospermia [11]. Some subfertile couples are seeking ART treatment due to STD infection.

Some studies report that the subfertile men with genitourinary infection caused by *C. trachomatis* had increased sperm DNA fragmentation [12], leading to sperm apoptosis [13] and low to sperm quality [10]. In animal experiments, *C. trachomatis* infection has been shown to inhibit oocyte transport in the mouse oviduct [14]. These studies suggest that *C. trachomatis* infection has the potential to negatively affect gamete formation and development. Despite the advances in knowledge about *C. trachomatis* infection, the impact of prior infection *C. trachomatis* on clinical outcomes after successful treatment is unknown. Therefore, the present study was carried out to evaluate *C. trachomatis* infection in subfertile couples I south China that were diagnosed by Taq-man PCR, and to deteremine whether the infection has any adverse effects on the clinical outcomes after treatment is completed.

## Methods

### Study population

The study patients were recruited from couples of reproductive age complaining of primary or secondary infertility that came to our reproductive center for ART treatment, from January 2010 to December 2014. For the study, a total of 30760 patients were screened for epidemiology statistical analysis. After fulfilling the eligibility criteria, 970 patients were diagnosed with *C. trachomatis* infection and 761 of these were included for clinical outcomes analysis. Subfertile (*n* = 1140) patients without *C. trachomatis* infection were recruited as the control group at corresponding period. This study was approved by the Research Ethics Committee of Shenzhen Zhongshan Urology Hospital, and informed consent was obtained from all couples prior to the study.

### Sample collection and *C. trachomatis* detection

A total of 30760 testing samples (including endocervical swab, semen and urethral swab) were obtained. Samples were collected into sterile containers and transported to the laboratory at room temperature within 2 hours. The DNA was extracted within 24 hours, according to instructions provided in commercial kit (Sansure Biotech, China). The endocervical and urethral swabs were placed in 1 ml normal saline; semen samples (50 μl) were added to 950 μl normal saline, and diluted (20×); the samples were sufficiently mixed by vortex for 15 s. Aliquots (0.5 ml) were centrifuged at 12000 rpm for 10 min, and the supernatants discarded. The pellets underwent lysis, mixed thoroughly and waited for 10 min in room temperature, and were stored at -20°Cuntil PCR analysis was performed. We performed four different concentration reference standards (from 10^4^ to 10^7^) to generate draw the standard curve, and then calculated the bacterial copy number for positive infection samples using absolute quantification real-time PCR methods. The kit for in vitro clinical diagnosis relies on absolute quantification of *C. trachomatis* burden (using FAM-labeled Taq-man primer–probe set), as assessed by the inclusion of known *C. trachomatis* standards. Furthermore, this system includes a Vic-labeled internal standard primer–probe set to test for the presence of PCR inhibitors. The PCR amplification was assessed using the ABI PRISM 7500 Sequence Detection System (Applied Biosystems, Foster City, CA). The assay set up internal control, positive control and negative control. The sample was deemed as positive if the number was higher than 200/ml copies.

### Ovarian stimulation protocol

All female patients received the luteal-phase gonadotrophin-releasing hormone agonist protocol. Since the pituitary was adequately suppressed (triptorelin acetate injection; Tiantaishan Pharmaceuticals, China), ovarian stimulation was induced with human menopausal gonadotrophin (Menotropins for Injection; Livzon, China) and recombinant FSH (Gonal-F; Merck Serono, Switzerland). Human chorionic gonadotrophin (hCG; Chorionic Gonadotrophin for Injection; Livzon, China) was injected when two or more dominant follicles reached 18 mm in diameter. Oocytes were collected 36 h after hCG administration by vaginal ultrasound guided follicular aspiration.

### Embryo culture, transfer and pregnancy evaluation

Fertilization was taken 16–18 h after IVF (day 1; oocytes retrieved day defined as day 0) by the presence of pro-nuclei. Zygotes were cultured in Quinn's Advantage Cleavage Medium (Quinn's 1026; SAGE, USA) containing 10% (v/v) serum protein substitute (SAGE). On day 3, embryos were scored using morphological criteria as previously described [15]. Grade 1 and 2 embryos were defined as high-grade embryos. Two or three cleavage-stage embryos (at least one high-grade embryo) were selected for transfer and the remaining embryos were stored in liquid nitrogen. The clinical pregnancy was identified by ultrasound examination at 5 weeks after the embryo transferr. The fertilization rates, cleavage rates, clinical pregnancy rates, implantation rates, miscarriage rates, take baby home rates and other data were collected and analyzed.

### Statistical analysis

Continuous data were presented as mean ± standard deviation (SD) and percentage. Mann-Whitney U test, Pearson's chi-squared or Fisher's exact tests were used to determine the difference. The statistical analyses were performed by SPSS 17.0 software (SPSS Inc, Chicago, IL, USA). *P* < 0.05 was considered statistically significant.

## Results

### Prevalence of *C. trachomatis* for subfertile patients

**Fig. 1:**
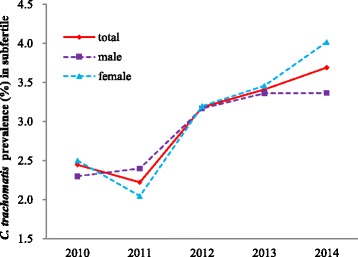
The incidence of *C. trachomatis* in subfertile individuals from 2010 to 2014

A total of 30760 samples were collected for the study over a period of five years and 970 patients identified as positive by PCR diagnostics, giving a prevalence rate of 3.15% (Fig. 1). The prevalence rate was 2.45% in 2010 and was 3.69% in 2014, showing an increasing trend year by year. The incidence was similar between the two genders (*P* > 0.05).

### Prevalence of *C. trachomatis* during different times of the year

**Fig. 2:**
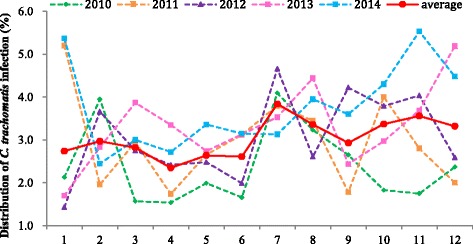
The prevalence and distribution of *C. trachomatis* infection in recent five years during different months

As shown in Fig. 2, the incidence of *C. trachomatis* was correlated with different seasons, in the past five years, the incidence was 3.40% in the second half year, it was slightly higher than the first half year (2.69%), and the difference was statistically significant (*P* < 0.05).

### Trails eligible for inclusion in clinical outcomes analysis

**Fig. 3:**
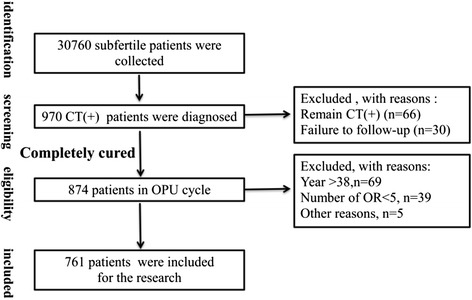
Schematic of the recruitment in the study. Abbreviation: *C. trachomatis*, Chlamydia trachomatis; OR, oocyte retrieve; OPU, oocyte pick-up

After identification, and eligibility screening, 761 patients (including single and dual infection of subfertile couples) were included in the study for clinical outcome statistical analysis, (Fig. 3). Meanwhile, 1140 subfertile couples without *C. trachomatis* infection were enrolled for the control group.

### Clinical outcomes in *C. trachomatis* infection

The basic parameters (Table 1), such as the age of couples and duration of infertility, were similar between the two groups (*P* > 0.05). The number of transferred embryos was 2.32 ± 0.59 in the *C. trachomatis* group and 2.25 ± 0.64 in control group, and the difference was statistically significant (*P* < 0.05). The implantation rate in *C. trachomatis* group was 32.84%, which was significantly lower than in control group (*P* < 0.05), which had a rate of 36.56%. The fertilization rate, cleaved rate, multiple pregnancy rate, clinical pregnancy rate, miscarriage rate and take-home baby rate were comparable between the two groups (*P* > 0.05).

**Table 1:** Parameters and clinical outcomes of patients with and without *C. trachomatis* infection

Variables	*C. trachomatis* (+)	*C. trachomatis* (-)	*P* value	OR (95% CI)
No. of couples	761	1140	/	
Male age (mean[IQR])	33.40 [29. -35]	33.79 [27-35]	0.874	
Female age (mean[IQR])	30.87 [24-32]	30.71 [25-32]	0.342	
Duration of infertility[IQR]	4.00 [2–5]	3.76 [2–5]	0.223	
Oocytes retrieved [IQR]	14.73[10–19]	14.68 [9–19]	0.529	
MII rate	88.80 (9957/11213)	88.82 (14873/16746)	0.966	0.998 (0.925-1.077)
Fertilized rate	76.55 (8584/11213)	75.98 (12724/16746)	0.271	1.032 (0.976-1.021)
Cleaved rate	96.28 (8265/8584)	96.03 (12219/12724)	0.348	1.071 (0.928-1.235)
Transfer cycles	643	938	/	
Transferred embryos[IQR]	2. 32 [2, 3]	2.25 [2–5]	0.036	
Embryo implantation rate	32.84 (490/1492)	36.56 (770/2106)	0.021	0.848 (0.738-0.976)
Clinical pregnancy rate	52.88 (340/643)	54.69 (513/938)	0.477	0.930 (0.760-1.137)
Miscarriage rate	13.24 (45/340)	16.18 (83/513)	0.238	0.790 (0.534-1.169)
Multiple pregnancy rate	43.82 (149/340)	46.78 (240/513)	0.395	0.887 (0.674-1.169)
Take baby home rate	45.88 (295/643)	45.95 (431/938)	0.978	0.997 (0.815-1.220)

*Note:* + = previously-positive *C. trachomatis*; *IQR* interquartile range

**Table 2:** Parameters and clinical outcomes in patients cured of *C. trachomatis* by gender

Variables	Male *C. trachomatis* (+)	Female *C. trachomatis* (+)	*P*	OR (95% CI)
No. of couples	374	387	/	
Male age (mean[IQR])	33.26 [30-36]	33.53 [29-36]	0.670	
Female age (mean[IQR])	30.77 [28-33]	30.97 [28-34]	0.916	
Duration of infertility[IQR]	4.00 [2–5]	4.01 [2–5]	0.180	
Oocytes retrieved [IQR]	14.50 [10–18]	15.00 [10-39]	0.420	
MII rate	87.72 (4756/5423)	89.60 (5201/5805)	0.002	0.828 (0.737-0.931)
Fertilized rate	76.29 (4137/5423)	76.61 (4447/5805)	0.689	0.982 (0.900-1.072)
Cleaved rate	96.74 (4002/4137)	95.86 (4263/4447)	0.032	1.280 (1.020-1.604)
Transfer cycles	307	336	/	
Transferred embryos [IQR]	2. 29 [2, 3]	2.35 [2, 3]	0.105	
Embryo implantation rate	34.14 (240/703)	31.69 (250/789)	0.314	1.118 (0.900-1.387)
Clinical pregnancy rate	54.72 (168/307)	51.19 (172/336)	0.370	1.152 (0.845-1.572)
Miscarriage rate	13.10 (22/168)	13.37 (23/172)	0.940	0.976 (0.521-1.828)
Multiple pregnancy rate	42.86 (72/168)	44.778 (77/172)	0.723	0.925 (0.603-1.420)
Take baby home rate	49.51 (152/307)	44.64 (150/336)	0.236	1.216 (0.892-1.659)

*Note:* + = previously-positive *C. trachomatis*

### Clinical outcomes in different genders

A total of 761 *C. trachomatis* infection patients were recruited in the study for clinical outcome analysis. These patients underwent successful treatment for the infections. They were further divided into two subgroups: male (374 patients) and female subgroup (387 patients) respectively. In the male subgroup, the MII rate was significantly lower but the cleaved rate was significantly higher, both differences were reached statistically significance (*P* < 0.05), when compared to the female subgroup. All other clinical parameters were not significantly different between genders (*P* > 0.05) (As shown in Table 2).

### Distribution and clinical outcomes stratified by age

**Fig. 4:**
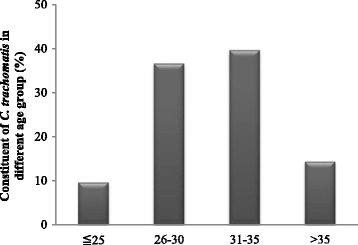
The distribution of *C. trachomatis* in different age groups

Patients were divided into four subgroups according to age: ≤25 years; 26-30 years; 31-35 years and >35 years. As shown in Fig. 4, the *C. trachomatis* constituent ratio different between the age groups. The age group having the highest prevalence rate was 31-35(38.12%), followed by 26-30 (30.22%), >35 (26.10%) and ≤25 years (5.56%). The basic parameters and clinical outcomes were comparable (*P* > 0.05) in different age brackets, even though the miscarriage rate was higher and the take-home baby rate were slight lower in advanced age group (*P* < 0.05) (Table 3).

**Table 3:** Parameters and Clinical outcomes of *C. trachomatis* in different age groups

Variables	≤25	26-30	31-35	35-38
No. of couples	73	278	301	109
Duration of infertility [IQR]	2.89 [2–5]	3.48 [2–6]	4.21 [2–6]	5.50 [3–8]
Oocytes retrieved [IQR]	16.71 [10–20]	15.8 [10–18]	13.94 [9–19]	13.03 [9–18]
MII rate	90.9 (1110/1220)	88.0 (3855/4378)	89.8 (3769/4195)	86.1 (1223/1420)
Fertilized rate	77.0 (940/1220)	76.9 (3371/4378)	76.8 (3223/4195)	73.9 (1050/1420)
Cleaved rate	95.9 (902/940)	96.4 (3250/3371)	96.2 (3103/3223)	96.1 (1010/1050)
Transfer cycles	67	223	253	101
Transferred embryos [IQR]	2.30 [2, 3]	2.20 [2, 3]	2.36 [2, 3]	2.50 [2, 3]
Embryo implantation rate	34.4 (53/154)	34.0 (167/490)	31.2 (186/595)	33.2 (84/253)
Clinical pregnancy rate	52.2 (35/67)	52.9 (118/223)	52.1 (132/253)	55.4 (56/101)
Miscarriage rate	14.2 (5/35)	7.6 (9/118)	12.1 (16/132)	28.5 (16/56)
Multiple pregnancy rate	51.4 (18/35)	41.5 (49/118)	40.9 (54/132)	50.0 (28/56)
Take baby home rate	44.7 (30/67)	48.8 (109/223)	45.8 (116/253)	39.6 (40/101)

### Distribution and clinical outcomes versus *C. trachomatis* copy number

**Fig. 5:**
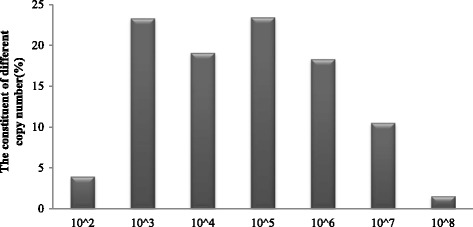
*C. trachomatis* DNA copies/ml in subfertile couples prior to treatment

The *C. trachomatis* copy numbers were mainly in the range of 10^3^ ~ 10^6^ copies (Fig. 5), accounting for approximately 80% of cases. Patients received ART treatment only after thereapy and the successful cure of infection. The longer the duration of infertility, the more embryos were transferred (*P* < 0.05), the other clinical parameters were similar among the groups (*P* > 0.05) (Table 4).

**Table 4:** Parameters and clinical outcomes for different *C. trachomatis* copy numbers

Variables	10^2^	10^3^	10^4^	10^5^	10^6^	10^7^	10^8^
No. of couples	30	177	145	178	139	80	12
Male age	34.50 ± 5.32	33.70 ± 4.73	33.59 ± 4.29	32.87 ± 4.28	33.59 ± 5.02	33.01 ± 4.12	31.83 ± 4.65
Female age	31.57 ± 3.95	30.92 ± 4.17	31.44 ± 3.87	30.22 ± 4.11	30.95 ± 3.89	30.88 ± 3.58	30.42 ± 4.74
Duration of infertility	3.07 ± 2.34	4.39 ± 3.05	4.04 ± 2.76	3.74 ± 2.72	4.12 ± 3.20	4.04 ± 2.54	2.50 ± 1.17
Oocyte retrieve	14.67 ± 6.00	15.28 ± 6.48	14.02 ± 6.10	15.05 ± 6.57	14.47 ± 6.18	14.71 ± 6.36	13.92 ± 5.65
MII rate	90.0 (396/440)	88.2 (2387/2705)	89.6 (1823/2033)	88.2 (2365/2679)	90.2 (1815/2012)	88.0 (1036/1177)	80.8 (135/167)
Fertilized rate	76.1 (335/440)	76.0 (2056/2705)	78.1 (1589/2033)	75.6 (2026/2679)	78.9 (1589/2012)	73.2 (862/1177)	76.0 (127/167)
Cleaved rate	97.9 (328/335)	96.6 (1987/2056)	96.9 (1540/1589)	95.9 (1943/2026)	95.5 (1519/1589)	95.4 (823/862)	98.4 (125/127)
Transfer cycles	20	156	125	145	123	63	11
Transferred embryos	2.10 ± 0.45	2.64 ± 0.51	2.30 ± 0.63	2.22 ± 0.60	2.20 ± 0.58	2.29 ± 0.61	2.36 ± 0.51
Embryo implantation rate	33.3 (14/42)	32.0 (128/400)	31.5 (91/288)	32.6 (105/322)	33.7 (91/270)	31.9 (46/144)	57.6 (15/26)
Clinical pregnancy rate	45.0 (9/20)	55.1 (86/156)	54.4 (68/125)	51.0 (74/145)	52.0 (64/123)	47.6 (30/63)	81.8 (9/11)
Miscarriage rate	11.1 (1/9)	10.4 (9/86)	14.7 (10/68)	10.8 (8/74)	15.6 (10/64)	15.2 (7/46)	0.0 (0/9)
Multiple rates	55.5 (5/9)	48.8 (42/86)	33.8 (23/68)	41.8 (31/74)	42.1 (27/64)	34.7 (16/46)	55.5 (5/9)
Take baby home rate	40.0 (8/20)	49.3 (77/156)	46.4 (58/125)	45.5 (66/145)	43.9 (54/123)	36.5 (23/63)	81.8 (9/11)

### Distribution and clinical outcomes of one-sided or two-sided infections

**Fig. 6:**
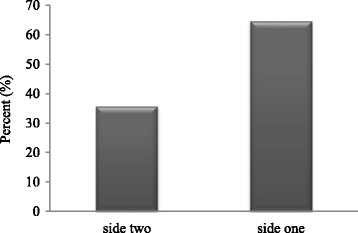
The constituent ratios of subfertile couples with *C. trachomatis* infection

For *C. trachomatis* positive couples, some were merely one-sided (one partner infected), while other couples had two sided infections (both partners infected). We found that on third of *C. trachomatis* cases were two-sided infections, and the remainder was one-sided. However, as long as the treatment was successful, all the clinical outcomes were not statistically different between the two groups (*P* > 0.05) (Table 5 and Fig. 6).

**Table 5:** Baseline demographics and clinical outcomes for different *C. trachomatis* copy numbers

Variables	Side two *C. trachomatis* (+)	Side one *C. trachomatis* (+)	*P* value	OR (95% CI)
No. of couples	271	490	/	
Male age	33.28[28-36]	33.46[30-36]	0.841	
Female age	30.87[27-34]	30.88[28-34]	0.996	
duration	4.04[2–5]	3.98[2–5]	0.828	
Oocyte retrieve	13.69[9–17]	15.31[11–19]	0.000	
MII rate	88.79 (3294/3710)	88.83 (6663/7501)	0.948	0.996 (0.879-1.128)
Fertilized rate	75.61 (2805/3710)	77.04 (5779/7501)	0.091	0.924 (0.842-1.013)
Cleaved rate	96.74 (2716/2805)	96.026 (5549/5779)	0.064	1.265 (0.986-1.622)
Transfer cycles	235	408	/	
Transferred embryos	2. 33[2, 3]	2.32[2, 3]	0.694	
Embryo implantation rate	31.08 (170/547)	33.86 (320/945)	0.270	0.881 (0.703-1.104)
Clinical pregnancy rate	51.49 (121/235)	53.92 (220/408)	0.552	0.907 (0.658-1.251)
Miscarriage rate	14.88 (18/121)	12.27 (27/220)	0.497	1.249 (0.657-2.375)
Multiple rates	40.50 (49/121)	45.00 (99/220)	0.422	0.832 (0.530-1.304)
Take baby home rate	43.83 (103/235)	47.06 (192/408)	0.429	0.878 (0.636-1.212)

## Discussion

Infertility is becoming an increasingly significant health problem in both developed and developing countries, undoubtedly, including China. The increase appears to coincide with the growing role played by sexually transmitted disease [16]. Here, we found that *C. trachomatis* infection has no adverse effect on clinical outcomes after *C. trachomatis* was thoroughly cured. This is one of the largest epidemiological studies of genital *C. trachomatis* in assisted reproductive technology patients thus far.

The prevalence of *C. trachomatis* in subfertile couples in this study was 3.15%, and increased yearly from 2.45% in 2010 to 3.69% in 2014. It is slightly lower than previous published reports, which showed varied prevalence rates of between 1% and 93.5% [17–19]. Discrepancies of the findings in these studies might be due to the fact that almost all of the sub-infertile patients in our study have a regular sexual partner, and were asymptomatic prior to the study. In other study, many subjects had symptoms, such as warts, ulcers, ectopy, lesions, erythema and discharge; some had more than one sexual partner. Other different between our study and others include the criteria and sample size of patients, area of residence, occupation, gender, diagnostic methods, specimen type and clinical study design.

The positive rate of *C. trachomatis* was 2.92% in males and 3.04% in females, respectively, which is slightly higher than previous reports [20]. The incidence in different genders is close [21], in contrast to other indications of higher rates in females than in males [22]. In these literatures, the subjects are mainly women involved in prostitution; females carry an increased risk for infection based on their anatomy as well. In contrast, the majority of patients in this study have a regular lifetime partner, thus the risk of sexually transmitted infections is relatively lower.

In the *C. trachomatis* positive group, the number of transferred embryos was significantly higher, while the embryo implantation rate was lower than the control group. A possible explanation is that the female age was higher and the duration was longer, even though these differences were not statistically significant (*P* > 0.05).

Some researchers have found that the prevalence of *C. trachomatis* is the highest in younger people especially those under the age of 25 years [23]. The highest prevalence rate in our study was in women aged 25-35 years, in males and females. However, the average age of patients in our center for ART treatment is about 32 years, and most patients were between 25 and 35 years old. Couples less than 25 years old seldom turn to ART treatment, unless there is a specific diagnosis reason of infertility. Advanced age females (>38 year) were excluded in the study as well, therefore the absolute number of patients ranging from 25 to 35 was proportionally high, resulting in a potential sources of bias. The miscarriage rate was 14.2% in those younger than 25 years and 28.5% in those older than 3 years. These results demonstrate that the best reproductive age was between 25 to 35 years old.

In our study, about one third of the couples were had two-sided infections and two thirds had one-sided infections; however, the clinical outcomes regarding the success of ART therapy are comparable in these two subgroups, suggesting that *C. trachomatis* infection had no adverse effect, regardless of whether the infection was present in one or both partners.

## Conclusion

*Chlamydia trachomatis* is common infection in subfertile people and it is critical to test for this organism before starting the ART process. The age range of infected individuals ranged between 25 to 35 years, and about one third of the couples had two sided infections that were transmitted sexually. For *C. trachomatis* infected patients, after a complete cure, there were no adverse effects on the clinical outcome, regardless of their gender, age group and number of copies.

## Abbreviations

ART: assisted reproduction technique
*C. trachomatis*: Chlamydia trachomatis
PID: pelvic inflammatory disease
SAGE: serum protein substitute
STD: sexually transmitted disease
